# Early Fire Detection Using Long Short-Term Memory-Based Instance Segmentation and Internet of Things for Disaster Management

**DOI:** 10.3390/s23229043

**Published:** 2023-11-08

**Authors:** Sharaf J. Malebary

**Affiliations:** Department of Information Technology, Faculty of Computing and Information Technology, King Abdulaziz University, P.O. Box 344, Rabigh 21911, Saudi Arabia; smalebary@kau.edu.sa

**Keywords:** instance segmentation, key-frame extraction, fire detection, IoT, disaster management

## Abstract

Fire outbreaks continue to cause damage despite the improvements in fire-detection tools and algorithms. As the human population and global warming continue to rise, fires have emerged as a significant worldwide issue. These factors may contribute to the greenhouse effect and climatic changes, among other detrimental consequences. It is still challenging to implement a well-performing and optimized approach, which is sufficiently accurate, and has tractable complexity and a low false alarm rate. A small fire and the identification of a fire from a long distance are also challenges in previously proposed techniques. In this study, we propose a novel hybrid model, called IS-CNN-LSTM, based on convolutional neural networks (CNN) to detect and analyze fire intensity. A total of 21 convolutional layers, 24 rectified linear unit (ReLU) layers, 6 pooling layers, 3 fully connected layers, 2 dropout layers, and a softmax layer are included in the proposed 57-layer CNN model. Our proposed model performs instance segmentation to distinguish between fire and non-fire events. To reduce the intricacy of the proposed model, we also propose a key-frame extraction algorithm. The proposed model uses Internet of Things (IoT) devices to alert the relevant person by calculating the severity of the fire. Our proposed model is tested on a publicly available dataset having fire and normal videos. The achievement of 95.25% classification accuracy, 0.09% false positive rate (FPR), 0.65% false negative rate (FNR), and a prediction time of 0.08 s validates the proposed system.

## 1. Introduction

Fire releases smoke, light, flames, heat, and chemical gases as a result of the combustion process [1]. Although fire has provided humans with prosperous living by providing the means for energy sources, heating, and cooking, uncontrolled fire can endanger properties and human lives. The National Fire Protection Association (NFPA) have reported 1.3 million fire cases in 2015, causing more than 3 thousand deaths and 15 thousand injuries [2]. Existing fire detection tools can be categorized into sensor-based, video-based, and hybrid techniques utilizing video-based sensors [3]. Sensor-based techniques utilize sensors to measure the levels of carbon dioxide, carbon monoxide, temperature, and smoke particles for efficiently detecting fire at an early stage [4]. The problem with these techniques is the cost and maintenance of these sensors. In contrast, video-based techniques utilize devices like cameras to capture the data and thermographic sensors to detect the fire pixel intensities [5]. The problem with these systems is their slow processing due to time taken during data collection, processing, and triggering the alarm in severe conditions [6]. This issue can be solved by adopting an efficient technique, which not only solves the processing time by reducing the dimensionality of extracted data, but effectively processes the input data in the lowest possible time. Thus, a need for reliable techniques to detect fire at an early stage is essential to prevent damage and the loss of human lives.

The domain of computer vision has been significantly transformed by the Internet of Things (IoT), specifically convolutional neural network (CNN) architectures. Cameras and sensors integrated into IoT devices enable the acquisition and transmission of vast quantities of visual data to the cloud for analysis. CNN models, which are widely recognized for their efficacy in object detection and image recognition, are crucial in deriving insightful information from this inundation of visual data. Deployed at the periphery or within cloud-based IoT platforms, these models execute environmental monitoring, anomaly detection, real-time surveillance, and facial recognition. By capitalizing on the potential of IoT and CNNs, it is presently feasible to develop sophisticated, interconnected systems capable of generating actions and decisions predicated on visual data analysis. This will undoubtedly improve safety, efficiency, and convenience across an extensive array of applications, ranging from smart cities to smart homes.

Connecting billions of smart devices creates the IoT, while an increase in the number of installed sensors has led to the emergence of the Tactile Internet (TI), which has various applications in the areas of e-health [7], smart surveillance [8], and disaster management [9]. Smart surveillance includes disaster and security management, where edge intelligence has a significant role. For rapid actions in disastrous situations, it is crucial to report unusual circumstances instantly. Disaster management mainly depends on fire/smoke recognition, which can be achieved using edge computing. Fire can spread due to human errors or system failures, which is a big risk for human lives and properties. In 2015, an overall damage of 3.1 billion USD was noticed as being caused by wildfire catastrophe, whereas in Europe, 10,000 km^2^ of fertile area is yearly affected from fire disasters [10]. Color-based fire detection methods [11] have a major issue with high rates of incorrect alarms. To overcome this issue, a hybrid approach was introduced using color, shape, and the motion characteristics of fire [12].

A well-performing and optimized approach is still a challenge, which should be optimally accurate, and have less complexity and a low false alarm rate. A small fire and the identification of a fire from a long distance are also challenges for state-of-the-art methods. In this article, a hybrid model for classifying and detecting fire images in real-time environments, called IS-CNN-LSTM, is proposed. A 57-layer CNN architecture with 21 convolutional layers, 24 rectified linear unit (ReLU) layers, 6 pooling layers, 3 fully connected layers, 2 dropout layers, and a softmax layer is proposed. Before training the CNN model, instance segmentation (IS) is performed to efficiently segment the fire. To reduce the training and testing duration of the proposed model, an algorithm to extract key frames based on the correlation between consecutive frames is proposed. Upon successful detection of the fire, alerts are sent to the connected IoT devices using the proposed network architecture, which will then lead to prompt actions and fewer casualties.

This article is structured as follows. Section 2 offers a literature review, while Section 3 describes the proposed classification and detection model, and its application to real-world problems. Using publicly accessible datasets, Section 4 demonstrates the effectiveness of the proposed model. In Section 5, the conclusion and prospective work are presented.

## 2. Literature Review

CNNs are widely used for fire identification problems [13]. CNNs have recently achieved efficient results on many domains including agriculture [14], medicine [15], and others [16,17,18,19,20,21,22,23,24,25]. A CNN-based fire detection method was proposed in [26], which was based on a limited dataset and not compared with any of the existing methods to prove their performance. Another CNN-based fire detection method utilizing the VGG16 and Resnet50 models was proposed in [27], which was trained and tested on a very small dataset of 651 images, and achieved an accuracy of 93%. Another CNN-based fire detection technique [28] was proposed to implement smart surveillance, which was trained on two-level datasets. The proposed model was huge in size (238 Mb), and constrained to deploy on restricted hardware systems. In [29], an optimized tradeoff between accuracy and false rate was maintained along with keeping the model size within accepted range. Moreover, a fire localization and detection network were proposed in [30] with a minimized model scope and false alarm rate, and a high accuracy.

Early fire detection systems were proposed with machine learning to analyze sensor data and fire images to attain precise accuracy. In [31], a You Only Look Once (YOLO)-based fire detection method was proposed, which was tested for flame recognition. The proposed model was learned on 196 images of fires and achieved an accuracy of 76%; however, the training images were not sufficient to fully saturate the model. In [32], a smoke detection method using a deep belief network (DBN) was proposed, where model was trained on 482 images and achieved 95% accuracy. Using the optical flow method, a neural network of deep convolutional long recurrent networks was tested for real-time fire detection and combustion detection [33]. A dataset containing 10,000 images and 70 video frames was used to train and test the proposed method, and achieved 93.3% accuracy. The results include the false detection of lights and flames as fire, which could be controlled by using other sensors. In [34], a fuzzy algorithm was put forth for the purpose of detecting fires using input from several sensors. A hybrid approach for fire detection using a fuzzy algorithm and CNN was proposed, which collected images from sensors and closed-circuit television (CCTV) [35]. In this system, CCTV images were first preprocessed using a CNN model to recognize fire; however, these CNNs were unable to identify fire in blind spots where cameras cannot be deployed. To remedy this issue, fuzzy logic computed the probability of fire presence by analyzing image and sensor data. This method was named S-FDS and was more flexible, as it used static as well as rule-based algorithms.

In recent times, object detection based on deep learning has gained prominence over sensor-based object detection. Park et al. [36] introduced the ELSTIC-YOLOv3 model as a means of identifying minute objects. Additionally, they discussed the dynamic fire tube, a distinctive feature of fire, in the same research article. In [37], a CNN-based model with an average precision accuracy of 98.7% was proposed by the research team. To detect objects, fast region-based convolutional neural networks (R-CNN) utilized high-quality region proposals generated by the end-to-end process-trained region proposal network (RPN) [38]. As they claimed in their paper [39], Liu, W. et al. introduced a single-shot detector (SSD) for multiple categories, that was substantially more accurate and faster than previous traditional works for single-shot detectors (YOLO).

Previously, many statistical techniques were used for data analytics. Each statistical algorithm can have unique characteristics based on its formula, the outcomes of its data analysis, and the algorithms to which it is related. Various machine learning (ML) techniques can also be applied to data analysis. ML techniques can be categorized as either superficial learning or deep learning. Superficial learning algorithms concentrate on superficial data structures, such as K-means clustering, decision trees, and SVM. In contrast, deep learning algorithms deal with deep layered structures which include CNN and deep neural networks [40]. In real environments, deep learning models have shown to be more flexible and expressive, compared with shallow learning models. End-to-end recognition is difficult for DNN, because it has limited abstraction ability, whereas CNN has a high abstraction power and can analyze the image features to examine the situation. At the beginning of a fire, the flame is of a small size and interval; in this situation, it is difficult to capture image features from flame video data [41]. Fuzzy algorithms utilize membership functions to represent proximity to situations that cannot be clearly divided. The environment affects the range of the membership method of fuzzy algorithms, whereas general-fuzzy algorithms disregard these variations. To overcome this limitation, an adaptive-fuzzy algorithm is introduced which can revise the membership method. These adaptive-fuzzy algorithms do not filter out exempted data because of errors in sensor data, thus affects result accuracy.

The single-shot multi-box detector (SSD) and YOLO are the finest illustrations of single-stage detection [42,43]. There are some restrictions to this form of detector. The significant class discrepancy between the foreground and background boxes has an impact on the accuracy of the predictions. The primary characteristics of single-stage detectors consist of a solitary feed-forward fully convolutional network that performs both the classification and recognition of the boundary boxes of objects. A deep learning object identification model was implemented on the Detectron2 platform to detect forest fires and the smoke plumes that accompanied them [44].

To address the constraints and simulate the distant interactions among input regions through the utilization of a self-attention mechanism—a fundamental component of transformers—transformers were suggested. In computer-vision tasks including video processing [45], image super-resolution [46], object detection [47] and segmentation [48], and image classification [49], transformers, namely, vision transformers (ViTs) [50] and DeiTs (data-efficient image transformers), demonstrated favorable performance. Two transformers based on vision, TransUNet and MedT, were implemented. Based on previous image transformers, the researchers developed two frameworks that were customized to their complex, unstructured environment. To evaluate these frameworks for forest fire segmentation, they utilized various backbones that were optimized. Self-attention offers three benefits in terms of fire pixel detection efficiency. Fewer parameters are present. The complexity of the model and the quantity of parameters are both diminished. Consequently, the required computational capacity is reduced even further, and the rate accelerates. Positive outcomes are possible when the attention mechanism is executed in parallel, akin to a CNN, since the calculation results of each phase are independent of those of the antecedent step. Emphasis should be placed on the pivotal aspects. Despite the length of the text or visual content, it is still possible to comprehend the essential points from the center without overlooking critical details. Generally, restricted attention can be directed towards critical information, thereby conserving resources, and expediting the acquisition of the most valuable data [51].

## 3. Proposed Work

**Figure 1:**
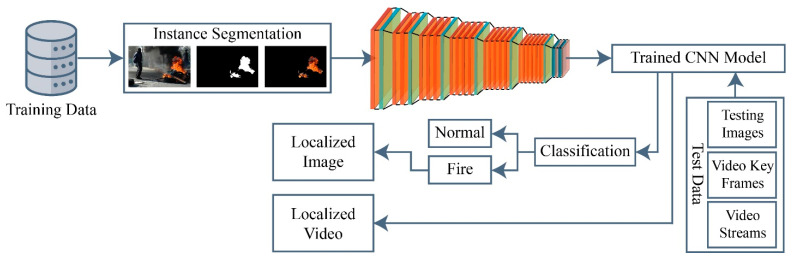
Schematic representation of the proposed method.

The early detection of fire becomes particularly challenging with factors like shadows, fire-like objects, and changing lights. Traditional local features are inadequate to detect a fire due to their low accuracy and high false negative rate. Extracting local features for fire detection is also a time-consuming and tedious task. These issues can be solved by extracting deep features using CNN models. After examining various pre-trained CNN models for target problems, a CNN model is proposed, which can classify and localize the fire at an early stage. Figure 1 depicts the schematic representation of the proposed method.

### 3.1. Instance Segmentation

**Figure 2:**
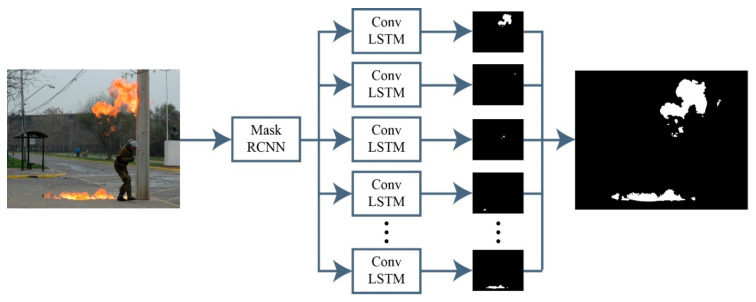
Structure of instance segmentation.

Semantic segmentation [52] is one of the most famous segmentation techniques, which deals with problems of known classes, where each pixel of image must belong to one predefined class and pixels are used to evaluate the predictions. But semantic segmentation cannot be applied to segment fire, as the instances of fire are unknown and have different shades and colors at different intensities. This problem is solved by employing instance segmentation, which is more challenging than other pixel-level techniques due to the nature of the solving of problems where classes are unknown. The evaluation of instance segmentation requires a loss function which is invariant to the assignment of pixels into different clusters. As instance segmentation is generally performed to count the objects in an image, it proves useful to count the instances of fire in an image. The approach proposed in [53] is inspired by the counting process followed by humans. Humans count objects by keeping track of accounted locations in an accurate spatial memory. Recurrent convolutional neural networks (RCNNs) were used to segment the objects while saving the current state in spatial memory. However, for the purpose of fire segmentation, the RCNNs did not perform well, as the fire instances are sometimes too small. A significant issue arises from the classification of fire instances as small objects, given that certain fire instances appear to be exceedingly minute in size in the images. Notably, the characteristics of these diminutive instances are comparatively fewer in number than those of the medium or large instances. These less specific characteristics complicate the task of RCNNs in detecting tiny instances. The features acquired subsequent to the convolution operation possess semantic information. Nevertheless, this intricate information that was concealed within the deep features is diminished during the pooling operation. To overcome this issue, the RCNN is replaced by Mask-RCNN, which provides improved results. The overall structure of instance segmentation utilized in this work is illustrated in Figure 2.

### 3.2. Deep CNN Architecture

**Figure 3:**
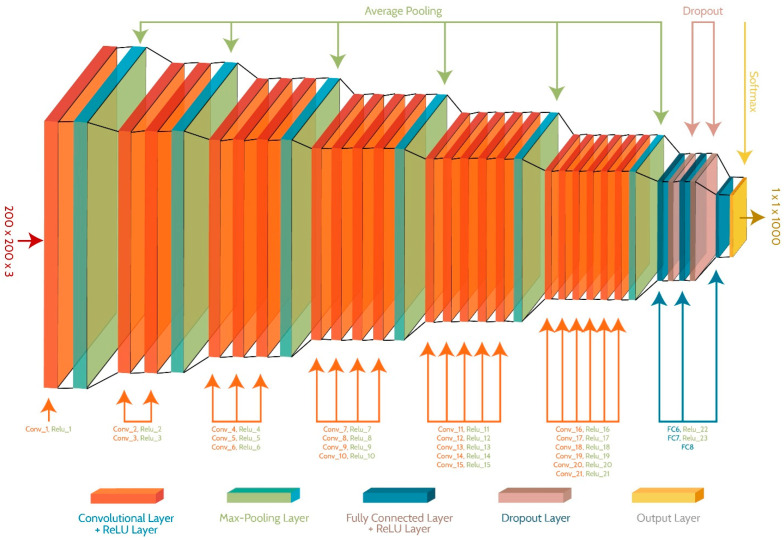
Architecture of the proposed CNN model.

A novel CNN model is proposed in this article, as the existing pre-trained models are trained on a large dataset, ImageNet [54], containing 1000 classes. The weights and activations of pre-trained networks are adjusted according to the images in the ImageNet dataset. These pre-trained models are structured in such a way that a single model can be utilized to classify multiple problems. This makes these networks too complex for classifying the simpler problems containing fewer classes. The parameters of our proposed network are updated by training it on fire and non-fire images only, which makes it more problem-oriented. The proposed network contains 57 layers, including 21 convolutional, 24 ReLU, 6 pooling, 3 fully connected, 2 dropouts, and a softmax layer. The network accepts an input of size 200 × 200 × 3, and the softmax layer provides 1000 features. The overall structure of the proposed model is shown in Figure 3. As the input images are already segmented, the activations on each layer remain consistent and gradually reduce. The purpose of this arrangement is to learn all possible features of fire along with different shades and intensities. The segmented images proved vital to train the network, and were capable of training a strong classifier and detector at the same time.

The structure of the CNN model is divided into 6 blocks, where each block increases the number of convolutional and ReLU layers by 1 and ends on an average pooling layer. The input is forwarded to block 1, where the combination of only convolutional and ReLU layers applies 96 filters of size 11×11 for generating 512 feature maps. Average pooling with a stride of 2 pixels is employed to shrink the size of the feature maps and retain the useful attributes by discarding the less important features. In the second block, 2 combinations of convolutional and ReLU layers apply 128 and 384 filters of size 5×5 and 3×3, respectively, and generate 256 feature maps. The average pooling of this block reduces the feature maps to 128. Blocks 3 to 6 contain 3, 4, 5 and 6 combinations of convolutional and ReLU layers, respectively, and apply a different number of filters to further convolve the input image. The average pooling of block 6 provides a descriptor map of size 64, which is forwarded to the fully connected layers, where layers FC6 and FC7 extract 5000 features, while FC8 extracts 1000 features. The Softmax layer provides 1000 features. A detailed overview of layers along with adjusted parameters is catalogued in Table 1.

### 3.3. Key Frames Extraction

The amount of video data collected from surveillance increases every day. Fire events rarely occur, and if a fire needs to be detected on a particular day or hour, it is still a tedious task to process and verify each frame from the video. If frames are extracted from a one-hour video at 30 fps, there will be 108,000 frames, and checking all these frames will take some serious amount of time. The execution and processing time reduces dramatically by extracting only key frames from a video. In this article, a method is utilized to extract only key frames by ignoring the duplicate frames. This is achieved by calculating the Pearson correlation coefficient (PCC) between two consecutive frames. Using the PCC to determine the correlation between two consecutive frames in an image or video sequence is a frequent practice. Denoted as “C,” the Pearson correlation coefficient quantifies the linear association between two sets of data. When examining consecutive frames, one may conceptualize each frame as a collection of data points (e.g., pixel intensities) and calculate the correlation between the two frames’ corresponding data points. The PCC is calculated by initially converting both frames into a grayscale, as grayscale images present the pixels as 2D arrays. If two consecutive frames are $F_{i}$ and $F_{i+1}$ with the same dimensions, and $F_{i}(a,b)$ and $F_{i+1}(a,b)$ denote their pixel values at position a and b, then the means of two matrices is calculated as

(1) $$\mu F_{i}=\frac{1}{P}\times \sum F_{i}(a,b)$$

(2) $$\mu F_{i+1}=\frac{1}{P}\times \sum F_{i+1}(a,b)$$

where P represents the total number of pixels, and $\mu F_{i}$ and $\mu F_{i+1}$ are the mean values of frames $F_{i}$ and $F_{i+1}$, respectively. After calculating the means, covariance is calculated as

(3) $$\varsigma =\frac{1}{P}\times \sum \left(\times \left(F_{i+1}\left(a,b\right)-\mu F_{i+1}\right)\right)$$

where ς is the covariance. The standard deviation for both frames is further calculated as

(4) $$\sigma F_{i}=\sqrt{\left(\frac{1}{P}\right)\times {\left(F_{i}\left(a,b\right)-\mu F_{i}\right)}^{2}}=\sqrt{\left(\frac{1}{P}\right)\times {\left(F_{i}\left(a,b\right)-\mu F_{i}\right)}^{2}}$$

(5) $$\sigma F_{i+1}=\sqrt{\left(\frac{1}{P}\right)\times {\left(F_{i+1}\left(a,b\right)-\mu F_{i+1}\right)}^{2}}$$

where $\sigma F_{i}$ and $\sigma F_{i+1}$ are the standard deviations. Finally, the PCC denoted by C is calculated as

(6) $$C=\frac{\varsigma }{\sigma F_{i}\times \sigma F_{i+1}}$$

**Figure 4:**
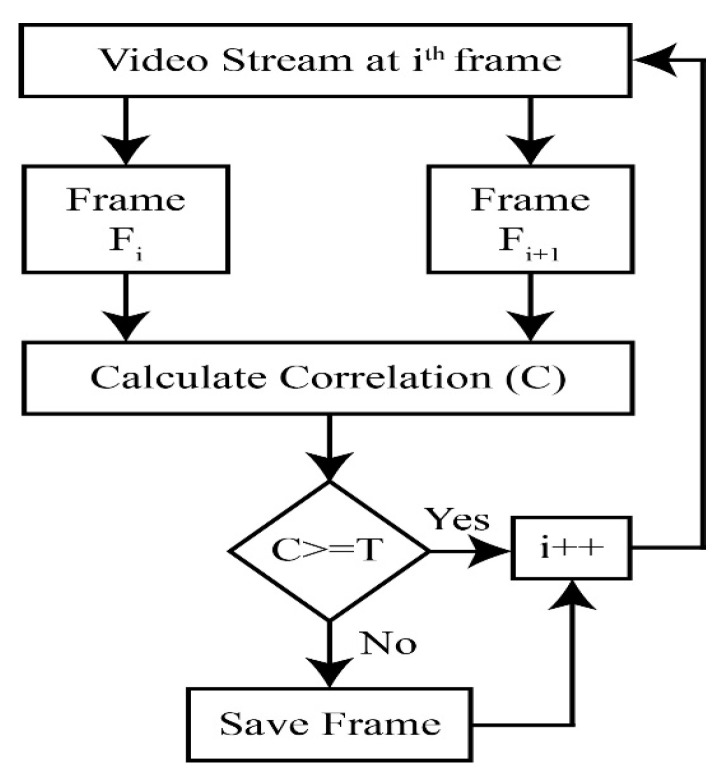
Flow diagram to extract key frames.

From Equation (6), C can have three possible values {0,−1,1}. The value of −1 denotes that the two frames do not have any similarity, 1 denotes that the frames are identical, while 0 indicates very little to no correlation. For the current task, C is checked against a threshold value T. If *C* is greater than or equal to *T*, the relationship is considered significant, and the images are similar; otherwise, the frames are considered as key frames. The overall flow of extracting key frames is explained in Algorithm 1. The flow diagram is also shown in Figure 4.
**Algorithm 1.** Extracting Key Frames from Video**Input:** A video stream**Output:** Key frames 1. $F_{i}\leftarrow$ All video frames2. $while\;(i<length(F_{i}))$3. $C=correlation(F_{i},\;F_{i+1})$4. if(C≥T) then    i++;  else    $SaveFrame(F_{i})$;    i++;**End**

### 3.4. Fire Classification and Localization

The proposed CNN architecture is designed to automatically learn robust features from raw fire data in both indoor and outdoor environments. Segmented fire images are provided as training data to label the test data as fire or normal images. This decision is based on the probability score of the CNN model. Once the fire and normal images are classified, the next step is to localize the fire within an image. Algorithm 2 describes the fire classification and localization process.
**Algorithm 2.** Classification and Localization of Fire**Input:** Trained classifier (*Classifier*), test data (*TD*), output type (*OT*), and trained CNN model (*Net*)**Output:** Localized fire images or video1. Analyze the input data (*ID*), either images (*I*) or video streams (*VS*)2. Analyze the *OT*, either localized image (*LI*) or localized video (*LV*)3. if(ID==I)
   Extract test features of ID and predict label using Net else if(ID==VS)    if (OT==LI)     Extract Key Frames      Repeat step 3    else if(OT==LV)     Resize video as per the Network Size      Localize the Video using Net4. Check the predicted Label   if (PredictedLable==Normal)
     No action required  else if(PredictedLable==Fire)
     Extract the features (FV) using layer FC7 of the CNN model.      Apply binarization using Threshold (T) as:      ${Image}_{Binary}=\left\{\begin{array}{c} 1,\;\;\;\;\;\;\;\;\;FV<T\\ 0,\;\;\;\;Otherwise \end{array}\right.$5. Localize the fire in the input image using ${Image}_{Binary}$

By sending test data to a trained classifier, which can be an image or a video stream, fire can be localized. Features are taken from the image or video frames, and their labels are predicted. Following the creation of a binary image utilizing the defined threshold and the predicted fire picture, the localization of the fire instances inside the image or video frame is subsequently accomplished.

### 3.5. Fire Analysis

At this point, the input images or videos containing fire are localized. The next step is to analyze the fire intensity and severity, as many post-fire assessments are based on this information. The intensity of the fire mainly depends upon the distance between the camera and the burning object. This distance is calculated by performing pre-processing steps like identifying all objects in an image, measuring the distance between the camera and the burning object, and measuring the area of the burning object. Objects are identified by training the proposed CNN model on a sub-part of a famous object dataset Caltech101 [55]. The selected part of the dataset contains 23 classes which can catch fire. The dimensions of these classes are preset to a default width and height. The other step of this analysis is to predict the severity of the fire for taking post-fire actions. Categorizing the fire level can determine whether to contact the house owner or the fire brigade. These fire levels are regarded as low-, moderate-, and high-severity. Algorithm 3 is used to determine the intensity of the fire and take the necessary post-fire steps.
**Algorithm 3.** Determining Intensity and Severity of Fire**Input:** Labelled Image**Output:** Alert concerning person/department1. Net← Trained Proposed CNN model on 23 classes2. $I_{i}\leftarrow$ Input Image3. $O_{i}\leftarrow \;$ Extracted objects from $I_{i}$ using Instance Segmentation4. $O_{f}\leftarrow \;DetectObjectOnFire(O_{i})$5. $Labeled_{O}\leftarrow IdentifyObjects\;(Net,\;O_{f})$6. ${Size}_{Actual}\;\left(w,h\right)\leftarrow FetchPresetSize(O_{f})$7. ${Size}_{Pixel\;}\left(w,h\right)\leftarrow CalculateLocalizedSize(O_{f})$8. ${Size}_{Predicted}\;\left(w\right)=\frac{{Size}_{Pixel}\left(w\right)}{{Size}_{Actual}\left(w\right)},\;\;{Size}_{Predicted}(h)=\frac{{Size}_{Pixel}(h)}{{Size}_{Actual}(h)}$9. $Dif\;\left(w\right)=\frac{{Size}_{Actual}\left(w\right)}{{Size}_{Predicted}\left(w\right)},\;\;Difference(h)=\frac{{Size}_{Actual}(h)}{{Size}_{Predicted}(h)}$10. if (Dif>1) then Object is Dif times bigger and each Dif pixels will be equal to 1 pixel$else\;if\;\left(Dif\leq 1\right)$ then Object is either equal or Dif times smaller and each 1 pixel will be equal to Dif pixels in case of smaller object11. ${Fire}_{Pixels}\;\left(w\right)\leftarrow CountFirePixels\left({Size}_{Pixel}\left(w\right)\right),$ ${Fire}_{Pixels}(h)\;\leftarrow CountFirePixels({Size}_{Pixel}(h))$12. ${Processed}_{FirePixels}\;(w)\;\leftarrow ProcessPixels({Fire}_{Pixels}\;(w),Dif\left(w\right))$ ${Processed}_{FirePixels}\;(h)\;\leftarrow ProcessPixels({Fire}_{Pixels}\;(h),Dif\left(h\right))$13. ${Fire}_{Effected\;}\left(w\right)=\frac{{Size}_{Actual}\left(w\right)}{{Processed}_{FirePixels}(w)}$, 
${Fire}_{Effected\;}\left(h\right)=\frac{{Size}_{Actual}\left(h\right)}{{Processed}_{FirePixels}(h)}$14. $Effected=mean\;({Fire}_{Effected}\left(w\right),{Fire}_{Effected}\left(h\right))$15. if (Effected≥60) then label fire as **High Severity**. else if (Effected≥15 and Effected<60) then label fire as **Medium Severity** else if (Effected<15) then label fire as **Low Severity**.

The magnitude of the fire instance is used as the basis for fire analysis. Instance segmentation is first carried out to detect fire items, after which the difference between the real and anticipated objects is determined. Fire pixels are calculated to accurately anticipate the fire severity after this difference has been calculated.

## 4. Experimental Results and Discussion

This section describes the investigations that were conducted to validate the proposed method. The information is described, including the experimental setup and the selected dataset. This dataset’s results are presented, followed by a comparison with existing techniques for fire detection and localization. Finally, a comprehensive discussion verifies the approach’s robustness and efficacy.

### 4.1. Experimental Setup

The proposed CNN model is trained using MATLAB 2022a on an NVIDIA GeForce GTX 1080 with an overall computation capability of 6.1, a clock rate of 1607-1733 MHz, and 7 multiprocessors. Stochastic gradient descent with momentum (SGDM) is the algorithm that represents the 64-minibatch training technique. The initial learning rate is fixed at 0.01 and decreased by a factor of 5 every 5 generations. Momentum is set to 0.7, and the utmost number of epochs is set to 150. Cross-entropy [41] is used as a suitable loss function because it has proven to be reasonable for many multiclass problems. The data is divided according to the standard proportions of 70-15-15 for training, testing, and validation, respectively.

### 4.2. Experimental Results

**Figure 5:**
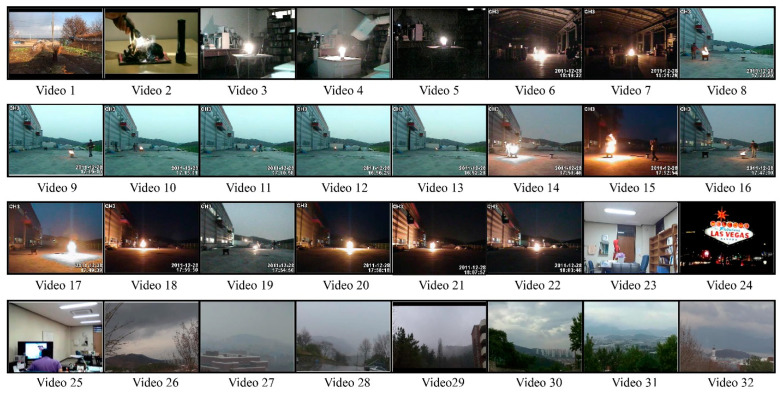
Sample videos from the dataset.

The publicly available dataset contains 32 videos, including 22 fire videos and 10 normal videos. The videos have a 24 fps rate, which makes a total of 64,049 frames of fires and 25,511 frames of normal images, and a grand total of 89,560 frames. The complexity, size, and background colors make this dataset challenging. The normal images contain fire-like objects, which makes the detection and classification even harder. Figure 5 illustrates a few test images, offering one frame each from all videos, while Table 2 presents a basic description for this dataset.

**Figure 6:**
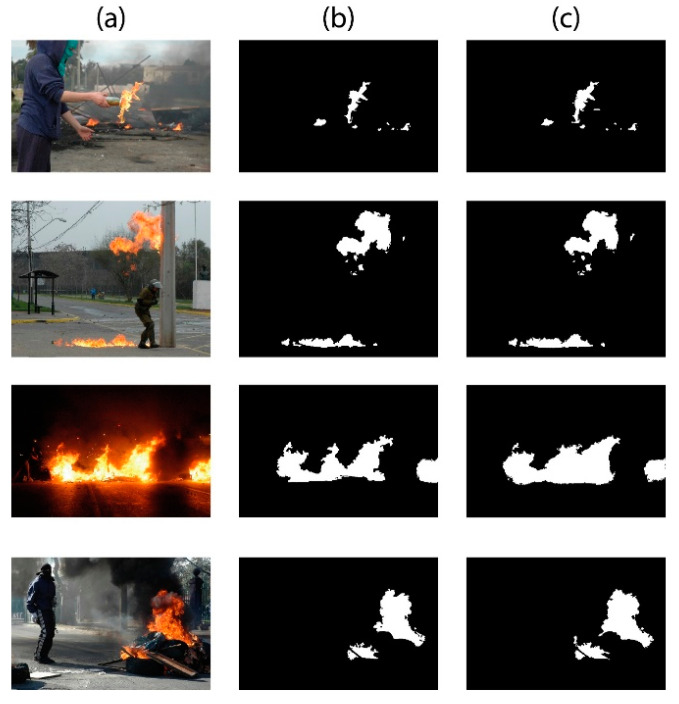
Results of instance segmentation. (**a**) Input image. (**b**) Ground-truth image. (**c**) Segmented image using instance segmentation.

**Figure 7:**
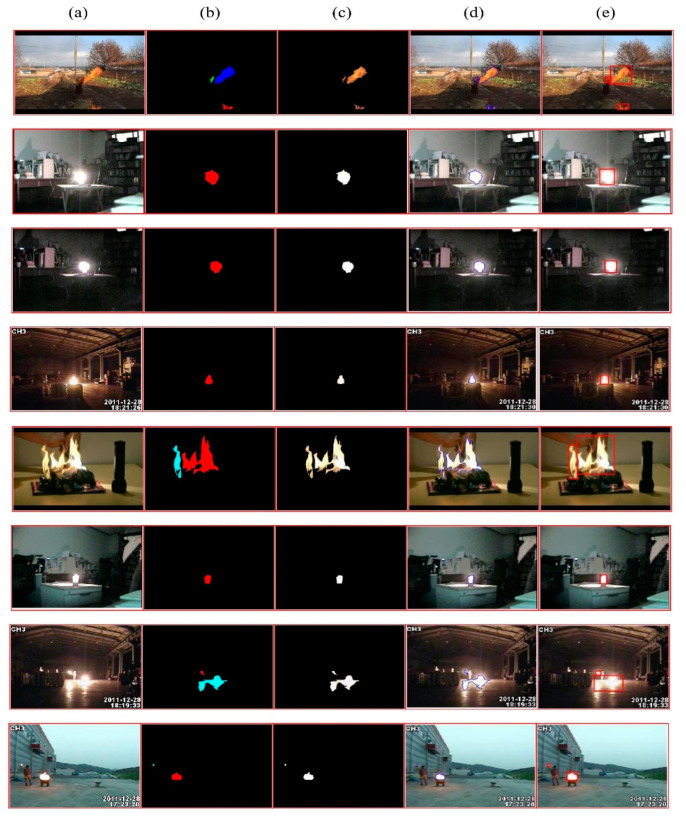
Results of the proposed model. (**a**) Original image. (**b**) Binary image extracted using instance segmentation. (**c**) Segmented image. (**d**) Boundary image, localized by detector. (**e**) Final predicted image.

In the proposed system, the initial instance segmentation proves vital as it helps the model to learn only fire features. The parameters of Mask-RCNN are learned using backpropagation. To prevent the effect of exploding gradient, gradients are clipped to make sure that each of their elements remains under the absolute value of 3. The Adam optimization algorithm [56] is applied to train the network by using an initial learning rate of 10^−4^ and reducing it by 0.1 of each error. As there was no overfitting during preliminary experiments, neither L2 regularization nor dropout was utilized throughout the segmentation process. The mini-batch size was set to 8 images per batch, and the initial weights of Mask-RCNN were randomly initialized within the range of [−0.04–0.04]. The results of instance segmentation on some sample images from a smaller dataset are presented in Figure 6, while the results of the proposed system are illustrated in Figure 7.

The proposed CNN model performs well on this dataset by maintaining a low false positive rate and high accuracy. The training time and prediction time are also noteworthy. Different experiments are performed including utilizing pre-trained models like AlexNet [57], InceptionV3 [58], and SuqeezeNet [59] before and after fine-tuning. All these networks are also serially used to note the impact. The proposed network is also experimented with before and after fine-tuning, as well as before and after adding the instance segmentation module. The outcomes of all these experiments are shown in Table 3. It can be clearly seen that the pre-trained models, fused models, and model without instance segmentation could not outperform the proposed model.

It is notable that the training time increases when instance segmentation is applied on the proposed approach, but the FPR and FNR rates are decreased to the minimum with the lowest prediction time of 0.08 s. The maximum accuracy is also noted at 95.25%, which is better than existing state-of-the-art techniques.

### 4.3. Robustness of Proposed Model

**Figure 8:**
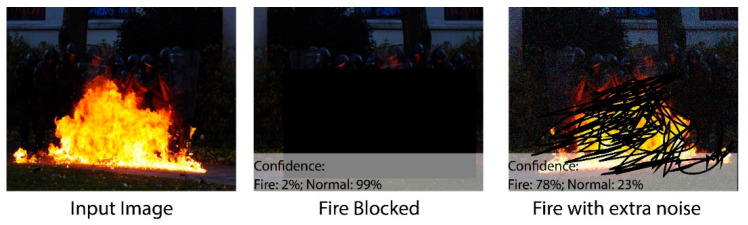
Robustness of the proposed model in different noisy conditions.

**Figure 9:**
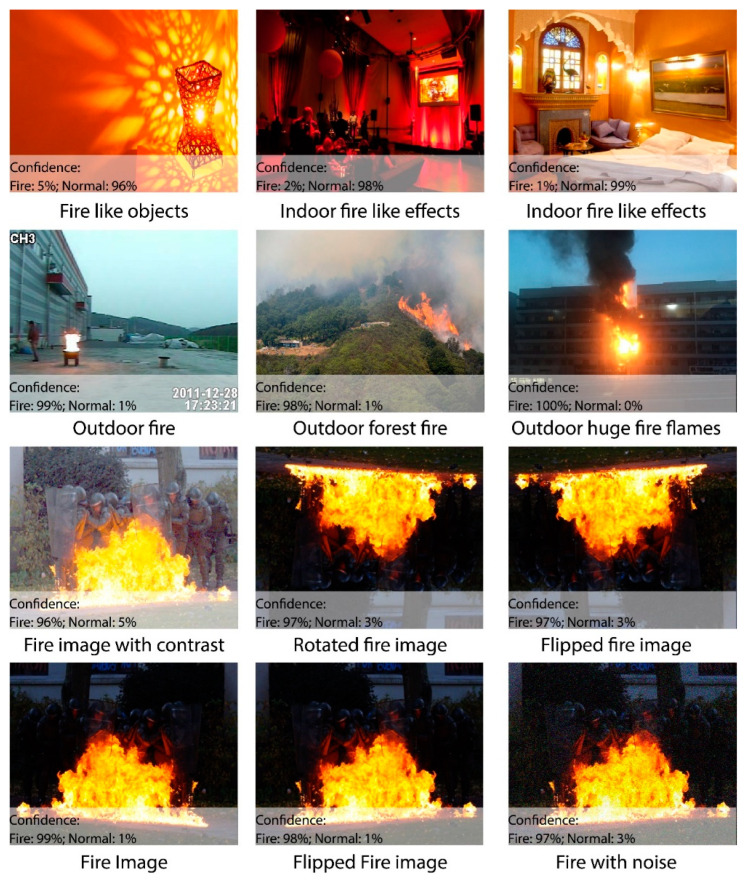
Output of the proposed model on fire as well as fire-like objects (first two rows). Output of the proposed model on different kinds of noise (last two rows).

**Figure 10:**
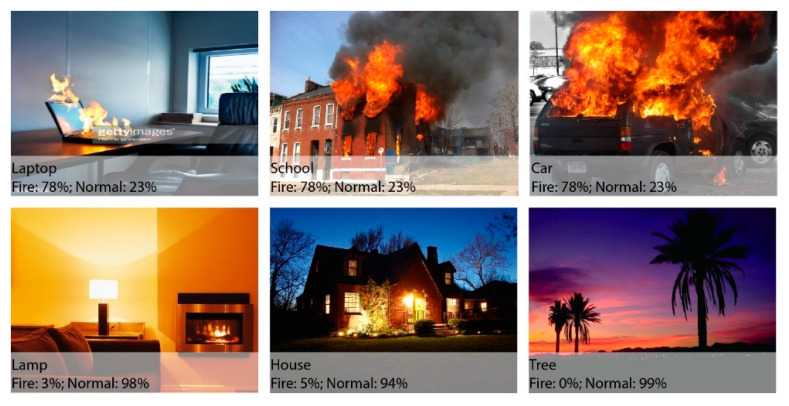
Output of the proposed model in different fire and non-fire scenarios.

The success of a fire detection system lies in its robustness against well-known attacks in uncertain environments. This section investigates the robustness of the proposed system by employing different attacks like fire-blockage and noise. Figure 8 shows that the proposed system performs well in most cases under uncertain environments and weather conditions. It can be clearly seen that the proposed system achieved efficient results on certain attacks. The fire analysis was carried out by testing images from the real-world, and it also achieved effective results. Figure 9 and Figure 10 show that the algorithm provides necessary information regarding the fire intensity and object on fire.

The device layer comprises cameras to obtain the image or video frame that requires fire detection. Images are initially stored in the local storage of the camera devices before being transmitted to the data engine, which performs the detection process. Multiple small cells (SCs) comprise 5G networks, each of which is linked to a distinct cache memory unit. The content providers (CPs) have reserved these cache memory units from the mobile network operators (MNOs). The multi-tenant environments provided by MNOs can store various types of data for a fee. To allocate the available cache memory slots to various Over-The-Top (OTT) CPs, a single optimized CP reserves an available cache memory slot in which various classes of content may be stored according to a specified spatial distribution of SC. This approach facilitates the determination of the minimal rate of missed caches in relation to the purchased slot. The prioritization of transmitted content over a 5G network is established based on several factors: demand rate, content availability, and popularity; concurrent content with comparable notoriety may also have an impact.

To optimize resources, the resource engine layer gains knowledge of network parameters such as data transit, communication quality, and network type. These optimizations are crucial for green communication in scenarios where bandwidth is limited, and large amounts of data must be transferred. In addition to ensuring the flexibility, scalability, and dependability of 5G systems, this layer optimizes hardware resources. The network functions that are executed in data centers via cloud infrastructure are connected to the hardware via the NFV. Intelligent programmable networks are tasked with the responsibility of facilitating a logical decoupling of network intelligence. This decoupling ultimately empowers applications to manipulate and request the services that the network provides.

**Figure 11:**
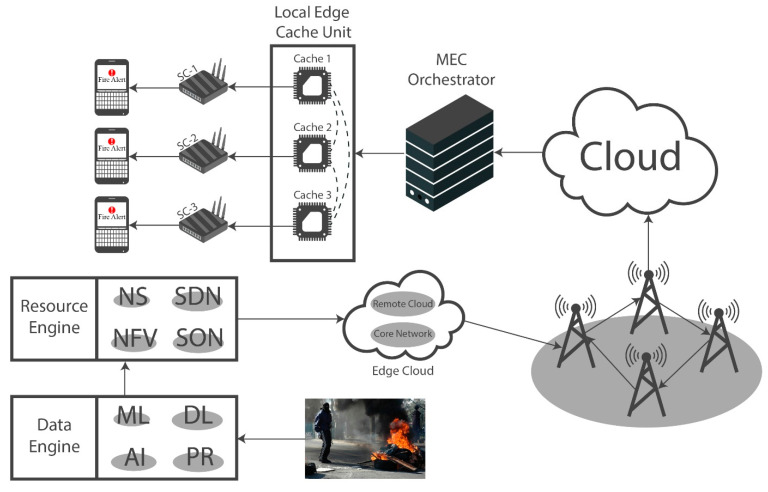
Architecture of alerting connected IoT devices on successful fire detection.

Then, 5G technology is utilized by the data engine layer to transmit images to the data centers. Machine learning and deep learning, which simulate human intelligence, are employed to acquire knowledge of features, and generate decisions regarding classification and detection. Figure 11 shows the overall architecture of detecting fire from input frames and alerting connected IoT devices in response.

To enable LSTM to reconcile minimal time lags exceeding one thousand discrete-time steps, constant error carousels implemented within special units enforce a constant error flow. Access is taught to and from multiplicative gate units in response to the constant error flow. LSTM has an O(1) computational complexity per time step and weight, and is local in both space and time. The pattern representations in our artificial data investigations are local, distributed, real-valued, and noisy.

### 4.4. Discussion

A system to detect fire at an early stage was proposed utilizing CNNs and IoMTs for disaster management, where a fine-tuned AlexNet model is used to detect fire with an accuracy of 94.39% and a false positive rate of 9.07% [60]. Many techniques have been proposed, which utilized the color shape and motion features and achieved an overall accuracy between 87 and 90% [61,62,63]. But these techniques proved vulnerable when fire-like objects were identified within the scene. In another technique, moving objects were initially detected to deal with the environmental changes throughout the timespan. These objects were then preprocessed by subtracting the background to extract the fire instances. The instances were evaluated based on color, shape, and difference between two consecutive frames in a video. The achieved accuracy of this technique was 95.55% with a false positive rate of 11.76% [64]. A transfer learning technique was implemented utilizing a pre-trained AlexNet network to detect the fire at an early stage. The proposed model was later fine-tuned using a SqueezeNet network, which reduced the size and feasibility of the approach to achieve an accuracy of 94.50% and a false positive rate of 8.87% [30]. Table 4 shows the experimental results along with a comparison to the previous techniques. The smart city framework proposed in reference [65] comprises four fundamental layers including cloud application, IoT, and fog layers. By integrating the IoT layer with fog and cloud computing, the proposed algorithm can gather and analyze data in real time. This facilitates quicker response times and mitigates the potential hazards to both human life and property. In terms of both precision and recall, the SFDS attained state-of-the-art performance, with a high precision rate of 97.1% across all classes.

In this work, a hybrid model is proposed, as shown in Figure 1, utilizing instance segmentation along with CNN architecture, as shown in Figure 2. The parameters of CNN are provided in Table 1, while the structure of the CNN model is shown in Figure 3. As the proposed model is trained and tested on video datasets, an algorithm is proposed, as shown in Figure 4 and explained in Algorithm 1, to extract key frames by calculating the correlation between consecutive pixels. After the extraction of key frames, the model is trained to classify and localize the fire in an image. Initially, the CNN model is trained on dataset to classify images, while the detector is trained on a subpart of a well-known dataset, Caltech-101. The detector provides information regarding the object on fire, while the fire is analyzed as per the proposed algorithm. The overall procedure of classification, localization, and fire analysis is explained in Algorithms 2 and 3. The description of the utilized dataset is given in Table 2, while Figure 5 shows some sample frames from each of the dataset videos. The results of instance segmentation are illustrated in Figure 6, while the detection and localization results are shown in Figure 7. Table 4 shows the classification results of different experiments. The robustness of the proposed model is checked against several attacks like injecting noise, blocking fire, rotation, and flipping operations. The achieved results are shown in Figure 8, Figure 9 and Figure 10. The model achieved better results than previously proposed state-of-the-art methods, and their comparison is presented in Table 4.

The performance of fire detection has been substantially improved by a 57-layer CNN model with IS for several compelling reasons. To begin with, a 57-layer CNN is capable of learning extraordinarily complex and discriminative features from input images due to the network’s depth. When applied to fire detection, these characteristics consist of complex patterns that are linked to regions of high temperature, smoke, and flames. By virtue of the network’s depth, these characteristics are automatically extracted, thereby enhancing its efficacy in distinguishing fire-related data from background noise.

Secondly, the integration of pixel-wise image segmentation and object detection in IS is especially advantageous in the context of fire detection. This functionality enables the model to accurately detect and demarcate specific fire occurrences within an image. In circumstances where multiple fire sources may be present, such as in a building with multiple rooms or compartments, this degree of granularity is crucial. By independently segmenting each fire instance, the proposed model enhances situational awareness and response by providing comprehensive information regarding the fire’s location and extent.

In addition, the depth of the 57-layer CNN model offers a robust capability for learning feature representations. Thus, the proposed model can adjust to an extensive variety of environmental conditions and fire-related scenarios. Solid generalizability across a wide range of data contributes to its robustness in practical fire detection scenarios. In addition to accurately delineating the perimeters of fire regions, the instance segmentation capability supports this by differentiating distinct objects despite their similar visual attributes. In general, by integrating instance segmentation and a deep CNN architecture, the model attains enhanced capability in accurately identifying and categorizing fires, thereby solidifying its position as a preeminent option for fire detection endeavors.

When designing a practical and responsive fire detection system, it is critical to prioritize the computational efficacy of a 57-layer CNN architecture utilized for instance segmentation and fire detection. Although the network’s profundity presents the possibility of capturing intricate image details, it can also result in heightened computational requirements. By implementing efficiency measures like model pruning, quantization, and optimized architecture, the computational complexity can be diminished without a loss of model precision. It is critical to implement hardware accelerators such as GPUs or TPUs when developing real-time applications. Additionally, bulk processing and efficient instance segmentation algorithms contribute to the optimization of the computational workflow. Attaining computational efficiency is critical for optimizing the utilization of available computational resources while ensuring the system’s ability to promptly respond to fire hazards and deliver dependable outcomes.

## 5. Conclusions

In this article, an automated system combining the properties of IS and CNN architecture is proposed to classify and detect fire in real-time environments. The CNN architecture is 57-layer deep, containing 21 convolutional layers, 24 ReLU layers, 6 pooling layers, 3 fully connected layers, 2 dropout layers, and a softmax layer. Training in CNN architecture is optimized by employing IS, which efficiently extracts the fire from images and video frames. To minimize the training and testing time of the proposed model, an algorithm is proposed to extract key frames based on the correlations between consecutive frames. The robustness of the proposed model is verified by testing it on real-time data, where the model achieved better results than state-of-the-art methods. This work can be implemented in real-world scenarios, like detecting fire in a supermarket or in a forest. As for future work, a CNN model with more depth can be utilized, and dimensionality can be reduced by implementing feature optimizing techniques. Key-frames can also be extracted by employing methods like genetic algorithm (GA) to improve the output of any model.

## Figures and Tables

**Table 1:** Detailed overview of layers along with adjusted parameters.

Combinations	Filters	Total Filters	Stride Size	Weight Size	Bias Vector	Activations
Input Layer	-	-	-	-	-	200×200×3
Convolutional + ReLU	11×11	96	[4×4]	11×11×3×96	1×1×96	512×512×96
Max Pooling	3×3	-	[2×2]	-	-	256×256×48
Convolutional + ReLU	5×5	128	[1×1]	5×5×48×128	1×1×128	512×512×128
Convolutional + ReLU	3×3	384	[1×1]	3×3×128×384	1×1×384	512×512×384
Max Pooling	3×3	-	[2×2]	-	-	256×256×192
Convolutional + ReLU	3×3	192	[1×1]	3×3×192×192	1×1×192	512×512×192
Convolutional + ReLU	3×3	128	[1×1]	3×3×192×128	1×1×128	512×512×128
Convolutional + ReLU	3×3	128	[1×1]	3×3×128×128	1×1×128	512×512×128
Max Pooling	3×3	-	[2×2]	-	-	256×256×64
Convolutional + ReLU	3×3	64	[1×1]	3×3×64×64	1×1×64	256×256×64
Convolutional + ReLU	3×3	128	[1×1]	3×3×64×128	1×1×128	256×256×128
Convolutional + ReLU	3×3	128	[1×1]	3×3×128×128	1×1×128	256×256×128
Convolutional + ReLU	3×3	256	[1×1]	3×3×128×256	1×1×256	256×256×256
Max Pooling	3×3	-	[2×2]	-	-	128×128×128
Convolutional + ReLU	3×3	128	[1×1]	3×3×128×128	1×1×128	128×128×128
Convolutional + ReLU	3×3	64	[1×1]	3×3×128×64	1×1×64	128×128×64
Convolutional + ReLU	3×3	128	[1×1]	3×3×64×128	1×1×128	128×128×128
Convolutional + ReLU	3×3	256	[1×1]	3×3×128×256	1×1×256	128×128×256
Convolutional + ReLU	3×3	128	[1×1]	3×3×256×128	1×1×128	128×128×128
Max Pooling	3×3	-	[2×2]	-	-	64×64×64
Convolutional + ReLU	3×3	512	[1×1]	3×3×64×512	1×1×512	64×64×512
Convolutional + ReLU	3×3	256	[1×1]	3×3×512×256	1×1×256	64×64×256
Convolutional + ReLU	3×3	128	[1×1]	3×3×256×128	1×1×128	64×64×128
Convolutional + ReLU	3×3	128	[1×1]	3×3×128×128	1×1×128	64×64×128
Convolutional + ReLU	3×3	96	[1×1]	3×3×64×96	1×1×96	64×64×96
Convolutional + ReLU	3×3	192	[1×1]	3×3×32×192	1×1×192	64×64×192
Max Pooling	3×3	-	[2×2]	-	-	32×32×96
FC6 + ReLU + Dropout	-	-	-	4096×25088	4096×1	1×1×4096
FC7 + ReLU + Dropout	-	-	-	4096×4096	4096×1	1×1×4096
FC8	-	-	-	1000×4096	1000×1	1×1×1000
Softmax	-	-	-	-	-	1×1×1000

**Table 2:** Basic description of the dataset.

Video Name	Original File Name	Resolution	Frames	Modality	Total Frames
Video 1	Flame1		402	Fire	64,049
Video 2	Flame2		411	Fire	
Video 3	Flame3		613	Fire	
Video 4	Flame4		373	Fire	
Video 5	Flame5		748	Fire	
Video 6	indoor_night_20m_heptane_CCD_001		1658	Fire	
Video 7	indoor_night_20m_heptane_CCD_002		3846	Fire	
Video 8	outdoor_daytime_10m_gasoline_CCD_001		3491	Fire	
Video 9	outdoor_daytime_10m_heptane_CCD_001		4548	Fire	
Video 10	outdoor_daytime_20m_gasoline_CCD_001		3924	Fire	
Video 11	outdoor_daytime_20m_heptane_CCD_001		4430	Fire	
Video 12	outdoor_daytime_30m_gasoline_CCD_001		6981	Fire	
Video 13	outdoor_daytime_30m_heptane_CCD_001		3754	Fire	
Video 14	outdoor_night_10m_gasoline_CCD_001		1208	Fire	
Video 15	outdoor_night_10m_gasoline_CCD_002		1298	Fire	
Video 16	outdoor_night_10m_heptane_CCD_001		3275	Fire	
Video 17	outdoor_night_10m_heptane_CCD_002		776	Fire	
Video 18	outdoor_night_20m_gasoline_CCD_001		5055	Fire	
Video 19	outdoor_night_20m_heptane_CCD_001		4141	Fire	
Video 20	outdoor_night_20m_heptane_CCD_002		1645	Fire	
Video 21	outdoor_night_30m_gasoline_CCD_001		6977	Fire	
Video 22	outdoor_night_30m_heptane_CCD_001		4495	Fire	
Video 23	smoke_or_flame_like_object_1		171	Normal	25,511
Video 24	smoke_or_flame_like_object_2		530	Normal	
Video 25	smoke_or_flame_like_object_3		862	Normal	
Video 26	smoke_or_flame_like_object_4		904	Normal	
Video 27	smoke_or_flame_like_object_5		8229	Normal	
Video 28	smoke_or_flame_like_object_6		7317	Normal	
Video 29	smoke_or_flame_like_object_7		2012	Normal	
Video 30	smoke_or_flame_like_object_8		849	Normal	
Video 31	smoke_or_flame_like_object_9		2807	Normal	
Video 32	smoke_or_flame_like_object_10		1830	Normal	
Total Frames					89,560

**Table 3:** Classification results of different experiments.

	Model	Fine-Tuning		Accuracy (%)	FPR (%)	FNR (%)	Training Time (s)	Prediction Time (s)
		No	Yes					
CNN Pre-Trained Models	AlexNet	✓		78.31	41.18	14.29	78.9	1.19
			✓	86.04	13.58	7.14	114.3	1.63
	InceptionV3	✓		83.87	29.33	10.65	69.8	0.83
			✓	87.56	7.22	2.13	93.4	0.94
	SqueezeNet	✓		74.39	14.67	7.80	63.5	0.98
			✓	84.77	9.41	5.50	87.4	1.23
	Fused	✓		89.47	11.76	9.74	397.2	0.78
			✓	90.35	5.88	1.50	247.9	0.63
Proposed	Without IS	✓		91.62	3.38	2.94	54.7	0.32
			✓	93.84	1.82	1.43	73.5	0.18
	With IS	✓		92.40	0.65	0.84	84.3	0.12
			✓	95.25	0.09	0.65	100.8	0.08

**Table 4:** Experimental results along with a comparison to the previous techniques.

Technique	FPR (%)	FNR (%)	Accuracy (%)
Rafiee [61]	17.65	07.14	87.10
Habiboğlu [62]	5.88	14.29	90.32
Chen [63]	11.76	14.29	87.10
Bellavista [60]	9.07	02.13	94.39
Foggia [64]	11.76	-	93.55
Muhammad [30]	8.87	02.12	94.50
Fernández [66]	-	-	92.6
Wahyono [67]	2.78	10.03	89.97
Talaat [65]	-	-	94.21
Proposed	0.09	00.65	95.25

## Data Availability

Publicly available datasets were analyzed in this study. This data can be found here: https://www.kaggle.com/datasets/phylake1337/fire-dataset.
